# Trusted Online Sources of Health Information: Differences in Demographics, Health Beliefs, and Health-Information Orientation

**DOI:** 10.2196/jmir.5.3.e21

**Published:** 2003-09-25

**Authors:** Mohan Dutta-Bergman

**Keywords:** Internet, source credibility, demographics, beliefs, health beliefs, health consciousness, consumer

## Abstract

**Background:**

The recent surge in online health information and consumer use of such information has led to expert speculations and prescriptions about the credibility of health information on the World Wide Web. In spite of the growing concern over online health information sources, existing research reveals a lacuna in the realm of consumer evaluations of trustworthiness of different health information sources on the Internet.

**Objective:**

This study examines consumer evaluation of sources of health information on the World Wide Web, comparing the demographic, attitudinal, and cognitive differences between individuals that most trust a particular source of information and individuals that do not trust the specific source of health information. Comparisons are made across a variety of sources.

**Methods:**

The Porter Novelli HealthStyles database, collected annually since 1995, is based on the results of nationally-representative postal-mail surveys. In 1999, 2636 respondents provided usable data for the HealthStyles database. Independent sample *t* tests were conducted to compare the respondents in the realm of demographic, attitudinal, and cognitive variables.

**Results:**

The most trusted sources of online health information included the personal doctor, medical university, and federal government. The results demonstrated significant differences in demographic and health-oriented variables when respondents who trusted a particular online source were compared with respondents that did not trust the source, suggesting the need for a segmented approach to research and application. Individuals trusting the local doctor were younger ( *t*_2634_= 4.02, *P*< .001) and held stronger health beliefs (F _1_= 5.65, *P*= .018); individuals trusting the local hospital were less educated ( *t*_2634_= 3.83, *P*< .001), low health information oriented (F _1_= 6.41, *P*= .011), and held weaker health beliefs (F _1_= 5.56, *P*= .018). Respondents with greater trust in health insurance companies as online health information sources were less educated ( *t*_2634_= 1.90, *P*= .05) and less health information oriented (F _1_= 4.30, *P*= .04). Trust in medical universities was positively associated with education ( *t*_2634_= 11.83, *P*< .001), income ( *t*_2634_= 10.19, *P*< .001), and health information orientation (F _1_= 10.32, *P*<.001). Similar results were observed in the realm of federal information credibility, with individuals with greater trust in federal sources being more educated ( *t*_2634_= 7.45, *P*< .001) and health information oriented (F _1_= 4.45, *P*= .04) than their counterparts.

**Conclusions:**

The results suggest systematic differences in the consumer segment based on the different sources of health information trusted by the consumer. While certain sources such as the local hospital and the health insurance company might serve as credible sources of health information for the lower socioeconomic and less health-oriented consumer segment, sources such as medical universities and federal Web sites might serve as trustworthy sources for the higher socioeconomic and more health-oriented groups.

## Introduction

With the rapid explosion of the Internet, one of the critical issues raised by experts involves the credibility of health Web sites [1]. This concern relates to the extent to which consumers are getting their information from Web sites that are not qualified to provide health information [2]. Practitioners and academics argue that source credibility lies at the heart of patient decision making in a medical context [1,2]. Trustworthiness and expertise of the source are the 2 critical criteria underlying source credibility judgments [3]. A source that is not trustworthy and does not have the expertise is more likely to mislead the patient, leading to misdiagnosis and mistreatment [2,3,4]. Whereas organizations such as the Centers for Disease Control and Prevention, National Institute of Health, nationally-recognized universities, and one's local doctor might qualify as trustworthy sources of health information, the exponentially-growing access to posting information on the World Wide Web also makes it possible for information to be posted by unqualified individuals and companies trying to sell their products to the public [5,6,7]. The important questions then are: How do patients make judgments about the credibility of Web sites? What sources do they consider to be most trustworthy?

These questions, although historically raised in speculative and prescriptive articles about the effects of the Internet on patients, have recently started receiving systematic empirical attention [2, 8-12]. Although the study of experts' perceptions of patient use of medical information on the Internet [13] is a worthwhile endeavor, it does not tap into the experiences of the patient. As a consequence, the discourse about consumer health-information searches on the Internet remains limited to the realm of the medical professional, reflecting the paternalistic sentiment of modern medical practice [14,15]. Based on the articulation that studying the health care consumer is central to the scholarship of Internet health information, this paper applies a consumer-based perspective to investigate the evaluation of credibility of health information on the Internet. It uses the HealthStyles data [16] to examine the differences in demographic, attitudinal, and cognitive variables between individuals on the basis of the different Internet sources of health information that they consider to be most credible.

## Methods

The Porter Novelli HealthStyles database, collected annually since 1995, is based on the results of 3 postal mail surveys. The initial survey, the DDB Needham Lifestyles survey (commissioned by DDB Needham Worldwide), is sent to a stratified random sample of approximately 5000 US adults in April of each year. The sample is generated from a panel of 500000 cooperating households that represent a range of sociodemographic characteristics. The second survey is a supplemental mailing of the Lifestyles survey to adjust the representation of particular households in the database. In 1999, the supplemental mailing was sent to 210 low-income households and 210 minority households to compensate for their lower return rates.

The third survey, HealthStyles, is sent to respondents who complete either the initial or supplemental Lifestyles survey. Respondents to each of the surveys are sent small gifts for their participation (such as a 20-minute calling card) and are entered into a cash prize drawing. In 1999, the response rate for Lifestyles survey was 68%. Of the Lifestyles respondents, 74% completed the HealthStyles questionnaire. The entire sample is weighted on age, sex, race/ethnicity, income, and household size to reflect the US Census population.

Usable data was provided by 2636 respondents. The sample was comprised of 48% men and 52% women. The mean age of the sample was 44.87 (SD = 16.71). The mean education level of the sample was 4.97 (SD = 1.29), and the mean household income of the sample was 12.59 (SD = 5.95).

### Measures

#### Credibility of Online Health Information

To measure the credibility of the different sources of health information, the following guideline was provided: "If you had to choose only three sources of health information on the Web, which three sources would you trust the most? ("X" only three)." Categories included "personal doctor," "local hospitals," "medical universities," "insurance companies," "community health organizations," and "federal government." Responses were measured in a dichotomous "Yes/No" format.

#### Demographics

Age was measured by a single item that simply asked the respondent to report his/her exact age in number of years. Education was measured by a single item, "education level of respondent." The scale ranged from 1 to 7, with 1 representing "attended elementary," 2 representing "graduated from elementary," 3 representing "attended high school," 4 representing "graduated high/trade school," 5 representing "attended college," 6 representing "graduated college," and 7 representing "post-graduate school." Income was measured by a single item "household income of respondent." The responses were measured on a 1 to 21 scale.

#### Health Consciousness

Health consciousness was measured by 5 items: "living life in best possible health is very important to me," "eating right, exercising, and taking preventive measures will keep me healthy for life," "my health depends on how well I take care of myself," "I actively try to prevent disease and illness," and "I do everything I can to stay healthy." Responses were measured on a 1 to 5 scale with 1 representing "strongly disagree," and 5 representing "strongly agree." When subjected to a principal component analysis with Varimax rotation, a single factor was produced with an eigenvalue of 2.36 and explaining 47.24% of the variance. The Cronbach's alpha for the scale was 0.72.

#### Health Information Orientation

Eight items were used to measure health information orientation. The items were: "I make a point to read and watch stories about health," "I really enjoy learning about health issues," "to be and stay healthy it's critical to be informed about health issues," "the amount of health information available today makes it easier for me to take care of my health," "when I take medicine, I try to get as much information as possible about its benefits and side effects," "I need to know about health issues so I can keep myself and my family healthy," "before making a decision about my health, I find out everything I can about this issue," and "it's important to me to be informed about health issues." Responses were measured on a 1 to 5 scale with 1 representing "strongly disagree" and 5 representing "strongly agree." A principal components factor analysis with Varimax rotation produced a single factor with an eigenvalue of 4.18. Factor loadings ranged from 0.62 to 0.82 and the factor explained 52.24% of the variance. Cronbach's alpha for the aggregated scale was 0.87.

#### Health-Oriented Beliefs

Health oriented beliefs were measured by 8 items. The respondents were provided the following instruction: "please rate each of the following health behaviors on a scale of 1 through 5 depending on how important you think that behavior is for your overall health." Items included "eating a diet that is low in fat," "eating lots of fruits, vegetables and grains," drinking plenty of water every day," "taking vitamins and mineral supplements regularly," "exercising regularly," "not smoking cigarettes," "not drinking alcohol or drinking in moderation," and "maintaining a healthy body weight." A principal components analysis with Varimax rotation yielded a single factor with factor loadings ranging from 0.52 to 0.77. Eigenvalue of the factor was 3.71 and it explained 46.31% of the variance. Cronbach's alpha for the aggregated scale was 0.82.

#### Analysis Plan

Data were analyzed in SPSS 10.00 for Windows (SPSS Inc). For the demographic comparisons of the individuals that trusted a particular source type with individuals that did not trust the source type, independent samples *t* tests were conducted. The reported *t* values for the demographic comparisons are 2-tailed. Since the health-oriented variables (health consciousness, health information orientation, and health-oriented beliefs) were correlated (Pearson's r ranging from 0.46 to 0.62), multivariate analyses of variance (MANOVA) were conducted for each source type.

## Results

The personal doctor emerged to be the most trusted source of online health information, followed by the medical university and the federal government. Of the respondents, 1548 (58.7%) reported trusting the personal doctor compared to 1088 (41.3%) respondents that did not list the primary doctor as one of the most trusted sources of health information on the Web. While 840 (31.9%) respondents documented their trust in the local hospital, 1796 (68.1%) respondents did not consider the local hospital as one of the most trusted sources of online health information. According to 1280 (48.5%) respondents, the medical university is one of the most trustworthy sources of health information on the Web compared to 1357 (51.5%) respondents who did not rate medical universities as one of the most trustworthy sources of online health information. Only 221 (8.4%) of the respondents reported considering the insurance company as one of the three most trustworthy sources of health information on the Web compared to 2415 (91.6%) respondents that did not consider the insurance company to be one of the most trustworthy sources. According to 979 (37.1%) respondents, community health organizations such as the American Cancer Society and March of Dimes were most trustworthy whereas 1657 (62.9%) respondents did not consider these sources to be among the most trustworthy. 1121 (42.5%) participants reported that they considered federal government resources such as the FDA, CDC, or NIH among the most trustworthy sources in contrast to 1516 (57.5%) individuals that did not consider the federal agencies to be trustworthy.

Participants who considered information provided by a personal doctor on the Web to be most trustworthy (mean = 43.77; SD = 16.44) were younger ( *t*_2634_= 4.02, *P*< .001) than participants who did not consider the online information provided by a personal doctor to be most trustworthy (mean = 46.42; SD = 16.97). No significant differences were observed in education and income. Furthermore, the results of the MANOVA (see Table 1) showed no significant effect of the trustworthiness of the personal doctor on health-oriented beliefs and attitudes (Wilk's = 1.00, F = 1.92, *P*= .12). Individuals who trusted online information provided by their local doctor (mean = 4.16; SD = 0.69) were more likely to hold stronger health beliefs as compared to those individuals that did not trust the online information provided by their personal doctor (mean = 4.09; SD = 0.68).

**Table 1:** Relationship between health-oriented variables and personal doctor as a trustworthy source

**Variable**	**F**	***df***	***P***	**^2^**
Health attitude	1.38	1	.241	0.001
Health belief	5.65	1	.018	0.002
Health information orientation	1.87	1	.171	0.001

Local hospitals often provide their information through Web sites. To what extent does local hospital trust as an online information resource vary with sociodemographics? Participants that trusted the local hospital as a Web resource were typically less educated ( *t*_2634_=3.83, *P*< .001) than their counterparts. They were also younger ( *t*_2634_=1.76, *P*= .08) than the respondents that did not trust the online information provided by the local hospital. The MANOVA (see Table 2) with the health-oriented dependent variables showed a significant main effect of local hospital trustworthiness on health-orientation (Wilk's = 1.00, F = 3.24, *P*= .02). Individuals who trusted online information provided by their local hospital (mean = 4.12; SD = 0.72 were less likely to hold stronger health beliefs as compared to those individuals that did not trust the online information provided by their personal doctor (mean = 4.18; SD = 0.64). They (mean = 3.66; SD = 0.76) also were less health information oriented than their counterparts (mean = 3.74; SD = 0.71).

**Table 2:** Relationship between health-oriented variables and local hospital as a credible source

**Variable**	**F**	***df***	***P***	**^2^**
Health attitude	0.86	1	.35	0.001
Health belief	5.56	1	.018	0.002
Health information orientation	6.41	1	.011	0.002

Comparisons of respondents in the context of their trust in medical universities point out that those individuals who trust medical universities as credible sources of online health information are younger ( *t*_2634_= 4.70, *P*< .001), more educated ( *t*_2634_= 11.83, *P*< .001), and have higher income ( *t*_2634_= 10.19, *P*< .001) than individuals that do not consider online information from medical universities to be credible. Results of the MANOVA (see Table 3) pointed out that the trustworthiness evaluation of the medical university had a significant main effect on health-oriented outcomes (Wilk's = 0.98, F = 14.52, *P*< .001). Participants with a greater degree of trust in the information provided by the medical university (mean = 4.21; SD = 0.32) held stronger health beliefs than their counterparts (mean = 4.07; SD = 0.72). Those who trusted online health information from medical universities (mean = 3.71; SD = 0.72) were also more health information orientated than their counterparts (mean = 3.65; SD = 0.73).

**Table 3:** Relationship between health-oriented variables and medical university as trustworthy source

**Variable**	**F**	***df***	***P***	**^2^**
Health attitude	0.06	1	.81	0.000
Health belief	25.81	1	.001< .001	0.010
Health information orientation	10.32	1	.001< .001	0.004

Insurance companies have recently ventured into the domain of providing online health information through their Web sites. Those individuals that considered insurance companies (mean = 4.81; SD = 1.18) to be most trusted sources of health information on the World Wide Web were less educated ( *t*_2634_= 1.90, *P*= .05) than the individuals that did not consider insurance companies to be most trusted sources of health information on the World Wide Web (mean = 4.97; SD = 1.30). Results of the MANOVA ) did not demonstrate a significant main effect of health-oriented variables. However, respondents who trusted health insurance companies (mean = 3.56; SD = 0.75) were less health information oriented than the respondents that did not trust the health insurance companies (mean = 3.70; SD = 0.73).

**Table 4:** Relationship between health-oriented variables and insurance company as a trustworthy source

**Variable**	**F**	***df***	***P***	**^2^**
Health attitude	0.11	1	.75	0.000
Health belief	0.00	1	.96	0.000
Health information orientation	4.30	1	.04	0.002

Participants reporting community health Web sites as most trusted resources were younger ( *t*_2634_= 8.93, *P*< .001), more educated ( *t*_2634_= 6.32, *P*< .001), and earned more ( *t*_2634_= 3.21, *P*< .001) than participants who did not trust community health organizations as most credible health resources. A significant main effect (Wilk's = 0.99, F = 10.36, *P*< .001) of community health organization trustworthiness was observed in the MANOVA (see Table 5). Respondents who considered community health Web sites as most trusted sources (mean = 4.21; SD = 0.60) held stronger health beliefs than their counterparts (mean = 4.09; SD = 0.73). They (mean = 3.76; SD = 0.68) were also more health information oriented than respondents who did not consider community health Web sites as credible (mean = 3.64; SD = 0.75).

**Table 5:** Relationship between health-oriented variables and community organization as a trustworthy source

**Variable**	**F**	***df***	***P***	**^2^**
Health attitude	0.28	1	.69	0.000
Health belief	10.02	1	002	0.004
Health information orientation	18.80	1	.001< .001	0.007

Federal agencies such as the National Institute of Health, National Cancer Institute and Center for Disease Control provide a great deal of health information to the public through their Web sites. In the next section, comparisons are drawn between individuals that consider federal Web sites to be most trusted sources of health information with individuals without a great deal of trust in health information provided by federal Web sites. Respondents considering federal Web sites to be most trusted sources of online health information were younger ( *t*_2634_= 9.84, *P*< .001) and more educated ( *t*_2634_= 7.45, *P*< .001) than respondents that did not consider federal Web sites as most trusted sources of online health information. However, no significant differences in income were observed. The MANOVA showed that the trustworthiness evaluation of a federal Web site had a significant effect on the health-oriented variables (Wilk's = 0.99, F = 6.50, *P*< .001). Individuals that trusted federal Web sites (mean = 3.72; SD = 0.73) were more health information oriented than individuals that did not trust federal Web sites (mean = 3.65; SD = 0.73).

**Table 6:** Relationship between health-oriented variables and federal government as a trustworthy source

**Variable**	**F**	**df**	***P***	**^2^**
Health attitude	2.92	1	.09	0.001
Health belief	0.10	1	.75	0.001
Health information orientation	4.45	1	.04	0.002

## Discussion

A recent guest editorial in the Journal of Medical Internet Research articulated the growing need for developing an adequate understanding of the information-use strategies of the online health consumer [17]. The article suggested that current debates over the issues of online health information quality within the expert domains [18] could only be resolved by opening up the discursive space to consumer-based approaches [16,17,19]. This study applied the consumer-based approach to study the trustworthiness of different sources of online health information. The central question answered in the current paper involved differences in demographics, attitudes, cognitions, and behaviors between individuals based on their trust in different sources of health information on the Web. The results demonstrated systematic differences among the different groups of individuals that trust different sources of online health information, voicing the need for a segmentation-based perspective in the realm of application and scholarship of online health information. Online health consumers are not a homogeneous entity and should not be treated as such in studies of source credibility [20]. Instead, they should be clustered into groups, and future scholarship on source credibility should be driven by this fundamental cognizance of individual-level differences in online health information behavior.

The results suggest that the personal doctor, medical university, and federal government Web site are the 3 most trusted sources of health information on the World Wide Web. These findings provide reason to be optimistic because the trustworthiness evaluations of patients do indeed mirror the trustworthiness suggestions and prescriptions of the medical profession [2]. In spite of the increasing consumer autonomy with the advent of the Internet, the personal doctor remains one of the most trusted sources of health information in the new-media environment, suggesting that more and more doctors need to explore the Internet as a viable medium for communicating with their patients.

The systematic differences between the different groups that trust different online health information sources have far-reaching implications for consumer-targeted health information delivery. For example, the findings that the online health information provided by local hospitals and insurance companies is more likely to be trusted by the unhealthy consumer segment suggest that these sources can be used as sites for Internet-based prevention campaigns targeting to change unhealthy behaviors. Local hospitals and insurance companies might be at an advantageous position for reaching this at-risk group with information on medical treatments. Health-oriented individuals who hold strong health oriented attitudes and health beliefs and are health information oriented, on the other hand, are more likely to trust information provided by medical universities, federal agencies, and community organizations (such as the American Cancer Society), suggesting that the trustworthiness judgments of higher socioeconomic groups are more closely aligned with the assessments of trustworthiness recommended by the existing expert-based literature on credible sources of health information. This match between expert opinions and higher socioeconomic groups perhaps articulates information gaps in society such that the higher socioeconomic groups have greater access to expert opinions than their lower socioeconomic counterparts.

The study has two important limitations. First, it uses secondary data, limiting further exploration of theoretically driven questions. Second, although the sources of health information surveyed in this study constitute a large portion of the available sources of health information on the Web, the study does not tap into all the different health information sources on the World Wide Web. Especially important to study are those online information providers that are driven by profit motives and pose potential threats to patient health. Future research needs to expand the findings of this study to other domains of health information sources such as pharmaceutical companies, individuals, and private organizations such as drkoop.com.
